# Functional characterization of NPM1–TYK2 fusion oncogene

**DOI:** 10.1038/s41698-021-00246-4

**Published:** 2022-01-18

**Authors:** Sudhakiranmayi Kuravi, Riley W. Baker, Muhammad Umair Mushtaq, Irfan Saadi, Tara L. Lin, Carolyn J. Vivian, Anusha Valluripalli, Sunil Abhyankar, Siddhartha Ganguly, Wei Cui, Kojo S. J. Elenitoba-Johnson, Danny R. Welch, Roy A. Jensen, Yogen Saunthararajah, Joseph P. McGuirk, Ramesh Balusu

**Affiliations:** 1 Division of Hematologic Malignancies and Cellular Therapeutics, Department of Internal Medicine, University of Kansas Medical Center, Kansas City, KS USA; 2 University of Kansas School of Medicine, Kansas City, KS USA; 3 The University of Kansas Cancer Center, Kansas City, KS USA; 4 Department of Anatomy and Cell Biology, University of Kansas Medical Center, Kansas City, KS USA; 5 Department of Cancer Biology, University of Kansas Medical Center, Kansas City, KS USA; 6 Department of Pathology and Laboratory Medicine, University of Kansas Medical Center, Kansas City, KS USA; 7 Department of Pathology and Laboratory Medicine, Perelman School of Medicine at the University of Pennsylvania, Philadelphia, PA USA; 8 Leukemia Program, Department of Hematologic Oncology and Blood Disorders, Cleveland Clinic Taussig Cancer Institute, Cleveland, OH USA

**Keywords:** T-cell lymphoma, T-cell lymphoma

## Abstract

Gene fusions are known to drive many human cancers. Therefore, the functional characterization of newly discovered fusions is critical to understanding the oncobiology of these tumors and to enable therapeutic development. NPM1–TYK2 is a novel fusion identified in CD30 + lymphoproliferative disorders, and here we present the functional evaluation of this fusion gene as an oncogene. The chimeric protein consists of the amino-terminus of nucleophosmin 1 (NPM1) and the carboxyl-terminus of tyrosine kinase 2 (TYK2), including the kinase domain. Using in vitro lymphoid cell transformation assays and in vivo tumorigenic xenograft models we present direct evidence that the fusion gene is an oncogene. NPM1 fusion partner provides the critical homodimerization needed for the fusion kinase constitutive activation and downstream signaling that are responsible for cell transformation. As a result, our studies identify NPM1–TYK2 as a novel fusion oncogene and suggest that inhibition of fusion homodimerization could be a precision therapeutic approach in cutaneous T-cell lymphoma patients expressing this chimera.

## Introduction

Gene fusions resulting from chromosomal rearrangements create chimeric proteins that play a significant role in the pathogenesis of various lymphomas and leukemias^1–3^. Deciphering the oncogenic biology of new chromosomal translocations is essential to understanding the molecular mechanisms of disease, which is vital to the development of therapeutic interventions^4–6^. NPM1–TYK2 (t; 5:19) is the first identified chimeric tyrosine kinase in 4% of CD30+ lymphoproliferative disorders (LPDs)^7^. Cutaneous CD30+ LPDs, the second most common type of cutaneous T-cell lymphoma (CTCL), include a clinicopathologic spectrum of benign lymphomatoid papulosis and primary cutaneous anaplastic large-cell lymphoma (ALCL)^8^. The fusion partners in this arrangement are the *nucleophosmin 1 (NPM1)* gene (5q35) and the *tyrosine kinase 2 (TYK2)* gene (19p13). NPM1 is a nucleolar phosphoprotein that performs diverse biological functions, including molecular chaperoning, ribosome biogenesis, DNA repair, and maintaining genomic stability^9,10^. TYK2 is a non-receptor tyrosine kinase that belongs to the Janus kinases (JAKs) family is associated with cytokine and growth factor receptors which activate STAT signaling^11,12^. NPM1–TYK2 is an 81 kDa chimera, comprised of the amino-terminal 257 amino acids of the NPM1 protein and carboxyl-terminal 461 amino acids length of the TYK2 protein, including the catalytic domain. The NPM1–TYK2 fusion consists of an almost full-length NPM1 protein except for the last 37 amino acids of the carboxyl-terminus, this contrasts with other NPM1 fusion proteins in which the NPM1 fusion partner is mostly limited to the oligomerization domain^13–15^.

A previous report documented an activated NPM1–TYK2 in vitro, but the characterization of the fusion protein was quite limited^7^. To understand the pathobiology of the fusion, it is essential to analyze the novel fusion kinase oncogenesis in relevant biological assays. In the present study, we focused on the functional characterization of NPM1–TYK2 utilizing both in vitro transformation assays and in vivo tumorigenicity in mouse xenograft models and detailed downstream signaling responsible for the oncogenicity. Our studies elucidate the oncogenic partnering role of NPM1 in the transformation potential of the NPM1–TYK2 fusion protein. Overall, our in vitro and in vivo models validate NPM1–TYK2 as a novel fusion oncogene and represent essential tools for future drug screening efforts to develop potential targeted therapies for CD30+ LPDs with this fusion event.

## Results

### In vitro transformation potential of the NPM1–TYK2 fusion gene

**Fig. 1:**
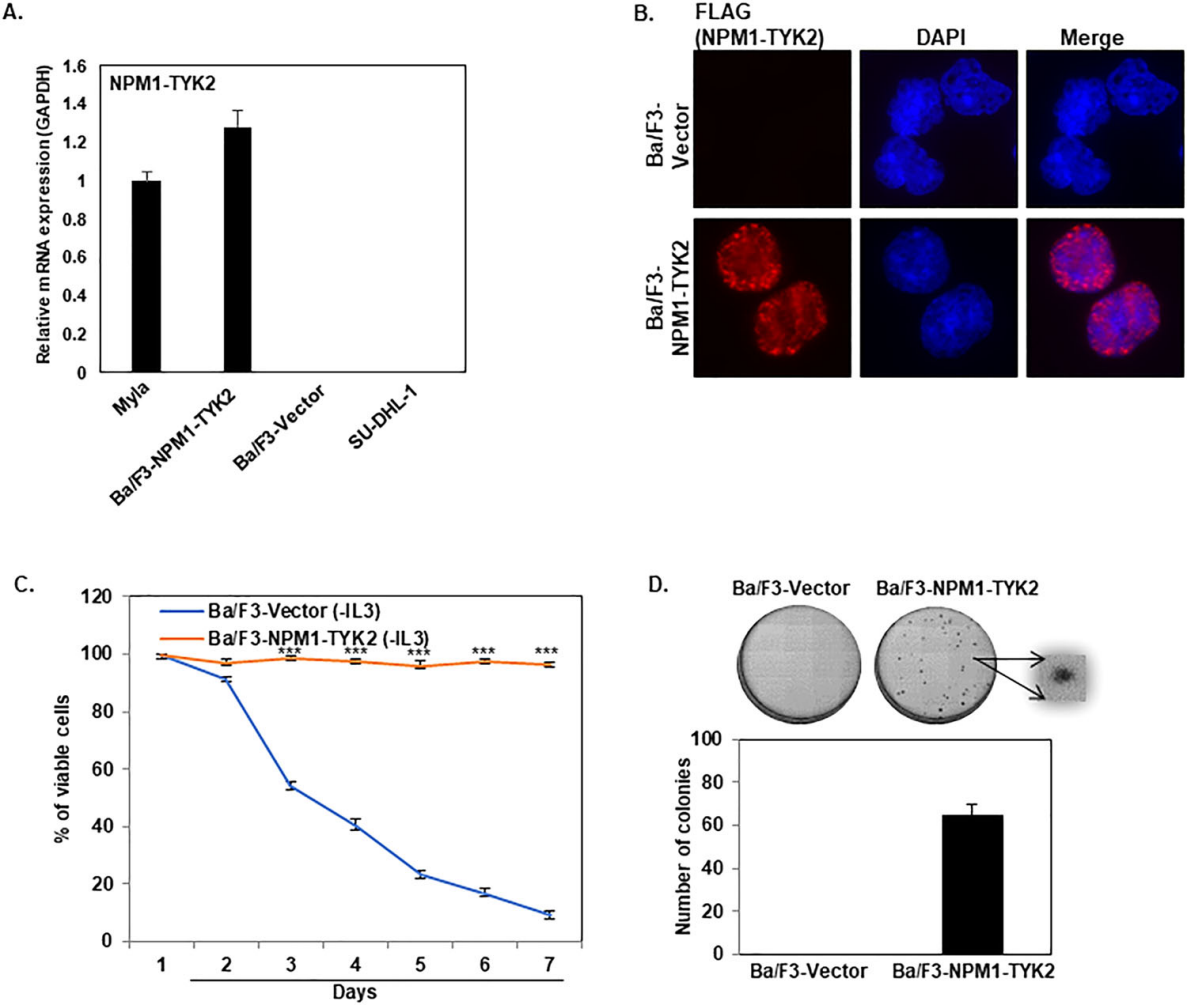
In vitro transformation potential of the NPM1–TYK2 fusion gene. **A** Detection of overexpressed NPM1–TYK2 mRNA transcript levels in transformed Ba/F3 cells by qRT-PCR. Myla and SU-DHL-1 cell lines were used as positive and negative controls, respectively. Columns represent the mean of three independent experiments; bars represent the SEM. **B** Subcellular localization of NPM1–TYK2. Ba/F3-Vector and transformed Ba/F3 (NPM1–TYK2) cells were cytospun onto glass slides, fixed, permeabilized, and stained for FLAG antibody and DAPI. Images were acquired with a fluorescent microscope using a 60× oil immersion lens. **C** Transformation of Ba/F3 cells to IL-3 independent growth. Ba/F3-Vector and transformed Ba/F3 (NPM1–TYK2) cells were grown in RPMI medium in the absence of IL-3. Cell viability from each condition was determined daily by trypan blue exclusion assay. Points represent the mean of three independent experiments; bars represent SEM. ^∗∗∗^*P* < 0.0005 was considered as statistically extremely significant. **D** The clonogenic potential of transformed Ba/F3 (NPM1–TYK2) cells. Ba/F3-Vector and transformed Ba/F3 NPM1–TYK2 cells were mixed with MethoCult medium and plated. After 7 days of incubation, the number of colonies was counted. Columns represent the mean of three independent experiments; bars represent the SEM.

The fundamental essay for establishing an oncogenic property of a new gene is the in vitro transformation assay^16^. The Ba/F3 in vitro transformation assay is a well-established method to determine the oncogenic potential of novel gene mutations in hematologic malignancies^17^. The Ba/F3 cell line requires IL-3 for normal proliferation and survival^18^. Oncogene overexpression in Ba/F3 cells allows for cytokine-independent growth through utilization of oncogene mediated survival signaling mechanisms^19^. The pCDH-EF1-MCS-T2A-Puro lentiviral expression plasmid was used to generate the overexpression constructs. pCDH-empty vector and pCDH-FG–NPM1–TYK2 lentiviral particles were made and transduced into Ba/F3 cells. Stable clones of the pCDH-empty vector (hereafter referred to as Ba/F3-Vector) and pCDH-FG–NPM1–TYK2 (hereafter referred to as Ba/F3–NPM1–TYK2) were selected using puromycin antibiotic selection. Once stable cell lines were established, we used total RNA from Ba/F3-NPM1–TYK2 cells to measure NPM1–TYK2 fusion transcript by real-time quantitative polymerase chain reaction (RT-qPCR). NPM1–TYK2 mRNA transcript levels in Ba/F3–NPM1–TYK2 cells were compared to Myla cells (i.e., the only available NPM1–TYK2 endogenously expressing cell line) while the SU-DHL-1 (NPM1-ALK+ ALCL) T-cell lymphoma cell line served as a negative control (Fig. 1A)^20^. We further examined the subcellular localization of the chimeric protein by immunofluorescence assay. As shown in Fig. 1B, our immunofluorescence data indicates NPM1–TYK2 fusion protein distribution in both nuclear and cytoplasmic compartments. To explore the oncogenic potential of the NPM1–TYK2 fusion gene, Ba/F3-Vector, and Ba/F3–NPM1–TYK2 cells were grown in the medium without IL-3 for seven days. As shown in Fig. 1C., vector control cells were unable to survive in the absence of IL-3 in the culture medium. In contrast, stable expression of NPM1–TYK2 in Ba/F3 cells promoted IL-3 independent growth utilizing NPM1–TYK2 mediated oncogenic signaling as a survival mechanism (Fig. 1C). Next, we examined the clonogenic potential of transformed Ba/F3–NPM1–TYK2 and Ba/F3-Vector cells by incubating each cell line in Methocult medium without IL-3 supplementation for seven days to allow colony formation. Ba/F3 cells expressing the NPM1–TYK2 fusion gene were able to produce colonies due to the oncogenic gain-of-function, while vector control cells could not survive in the absence of IL-3 growth factor (Fig. 1D). Thus, our in vitro transformation assay results with Ba/F3 cells demonstrate the oncogenic potential of the NPM1–TYK2 gene.

### Intracellular signaling that governs lymphoid cell transformation

**Fig. 2:**
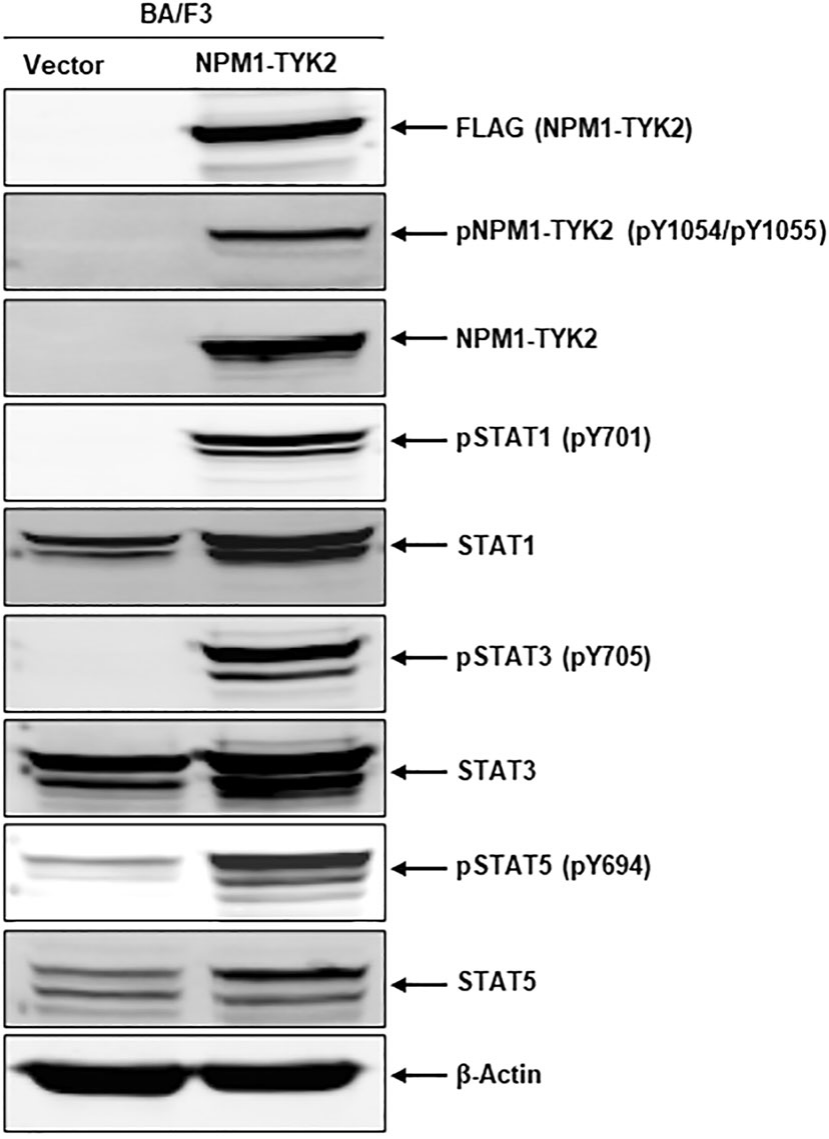
Fusion kinase NPM1–TYK2 oncogenic signaling drives the lymphoma cell transformation. Ba/F3-Vector and transformed Ba/F3–NPM1–TYK2 cell lysates were made, subjected to immunoblot analysis for NPM1–TYK2 (FLAG), phospho-NPM1–TYK2, phospho-STAT1/3/5, and respective total proteins. β-Actin served as an internal control.

Cancer cells acquire transformation potential through upregulation of survival signaling pathways resulting from oncogenic genetic alterations^21^. Once we confirmed the oncogenic potential of NPM1–TYK2, we investigated the critical signaling pathway responsible for fusion kinase-driven oncogenicity. In several cancers, TYK2 gene mutations result in constitutive activation of TYK2 kinase leading to upregulation of downstream STAT signaling^22,23^. Therefore, we examined Ba/F3–NPM1–TYK2 cells for fusion kinase activation and downstream STAT signaling. Our Western blot analysis data clearly indicate the upregulation of phosphorylated NPM1–TYK2 in transformed Ba/F3–NPM1–TYK2 cells. Activation of NPM1–TYK2 fusion kinase resulted in phosphorylation of downstream signaling molecules STAT1, STAT3, and STAT5 (Fig. 2). In contrast, Ba/F3-vector cells showed no activation of fusion kinase or downstream STAT signaling (Fig. 2). Collectively, our NPM1–TYK2 fusion kinase signaling results indicate that Ba/F3–NPM1–TYK2 cells acquired transformation potential through constitutive activation of fusion kinase and downstream STAT signaling.

### NPM1–TYK2 fusion kinase activation is an oncogenic driver in lymphoid cell transformation

**Fig. 3:**
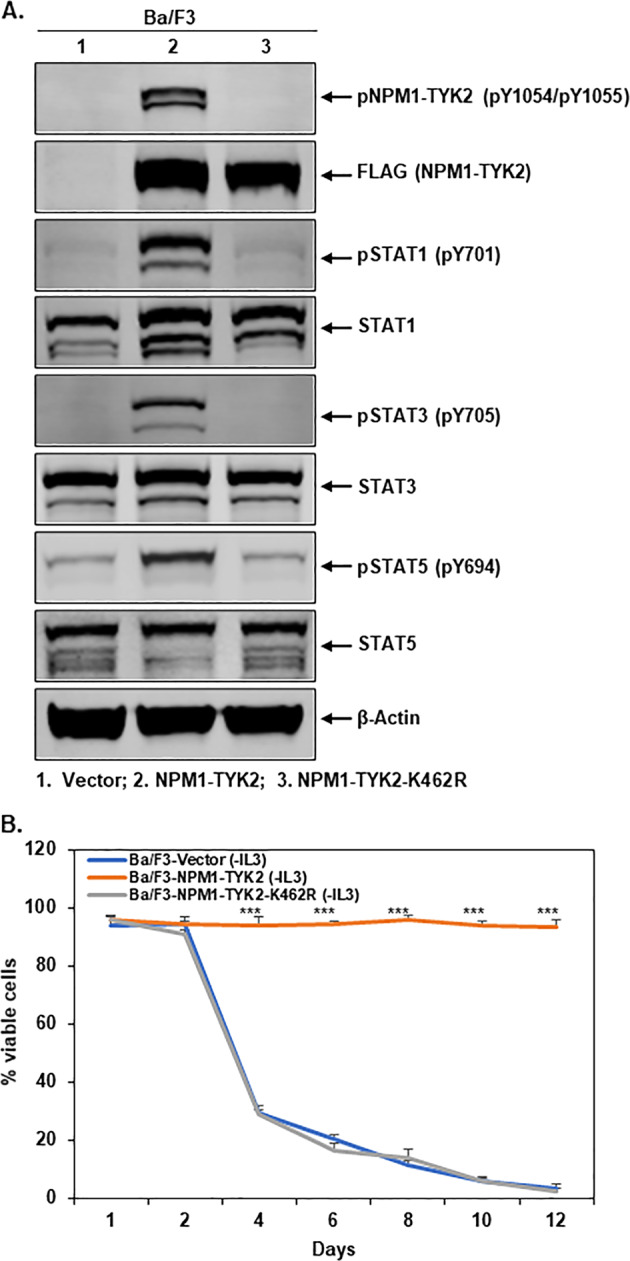
NPM1–TYK2 fusion kinase is an oncogenic driver in lymphoid cell transformation. **A** Fusion kinase-dead mutant blocks kinase activation and downstream signaling. Cell lysates were made from Ba/F3-vector, Ba/F3–NPM1–TYK2, and Ba/F3–NPM1–TYK2–K462R cell lines, and immunoblot analysis was performed for FLAG (NPM1–TYK2), phospho-NPM1–TYK2, phospho-STAT1/3/5, and respective total proteins. β-Actin served as an internal control. **B** Fusion kinase signaling drives cell transformation. Ba/F3-Vector, Ba/F3–NPM1–TYK2, and Ba/F3–NPM1–TYK2–K462R cells were grown in RPMI medium in the absence of IL-3. Cell viability from each condition was determined daily by trypan blue exclusion assay. Points represent the mean of three independent experiments; bars represent SEM. ^∗∗∗^*P* < 0.0005 was considered as statistically extremely significant.

To further validate the fusion kinase-driven transformation of Ba/F3 cells, we developed a stable Ba/F3–NPM1–TYK2–K462R kinase-dead mutant cell line. Cell lysates were made from Ba/F3-vector, Ba/F3–NPM1–TYK2, Ba/F3–NPM1–TYK2–K462R cell lines, and Western blot analysis was performed for phospho-NPM1–TYK2, phospho-STAT1/3/5, and respective total proteins. Ba/F3-NPM1–TYK2 and Ba/F3-NPM1–TYK2–K462R cells showed ectopic overexpression of FLAG (NPM1–TYK2) protein, whereas no expression was observed in Ba/F3-vector cells (Fig. 3A). Furthermore, wild-type fusion kinase expressing Ba/F3–NPM1–TYK2 cells showed activated fusion kinase and upregulation of downstream STAT1/3/5, whereas no activation of kinase was observed in Ba/F3-vector and Ba/F3–NPM1–TYK2–K462R cells (Fig. 3A). We also examined the IL-3 independent growth and transformation potential of the NPM1–TYK2 kinase-dead mutant compared to wt-NPM1–TYK2 and vector control cells. Ba/F3 vector and NPM1–TYK2 kinase-dead mutant cells were unable to survive without IL-3 in the medium, however, Ba/F3 cells expressing wt-NPM1–TYK2 cells showed IL-3-independent growth (Fig. 3B). The lack of signaling and cell viability in the absence of IL3 of fusion kinase-dead mutant cells clearly demonstrates that the NPM1–TYK2 fusion kinase activity is the oncogenic driver responsible for Ba/F3 cell transformation.

### In vivo tumorigenic potential of the NPM1–TYK2 fusion gene

**Fig. 4:**
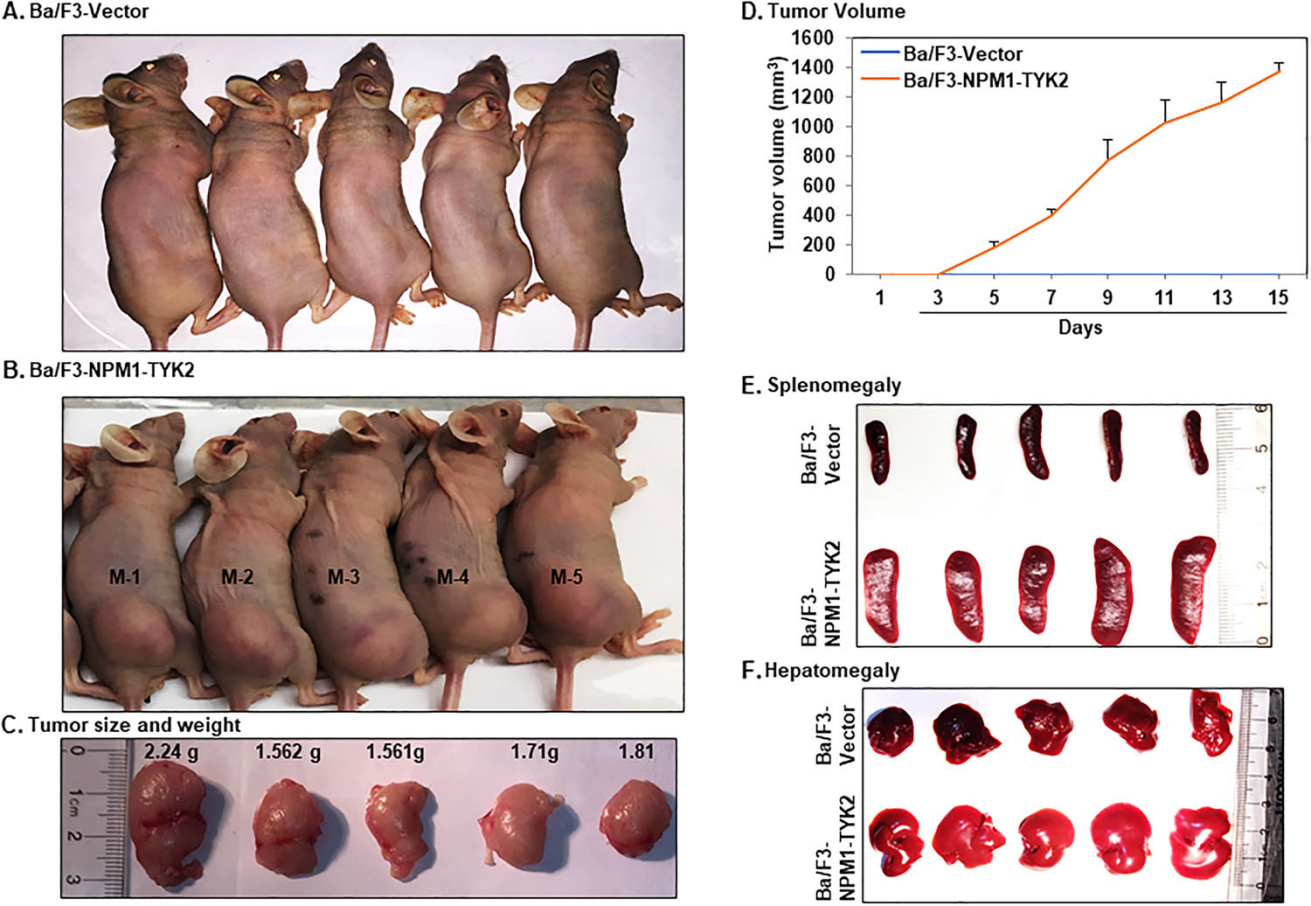
In vivo tumorigenic potential of the NPM1–TYK2 fusion gene. **A** Representative image of mice (*n* = 6) subcutaneously injected with Ba/F3-Vector showed no tumors. **B** Representative images of mice (*n* = 6) subcutaneously injected with transformed Ba/F3–NPM1–TYK2 show tumorigenesis. **C** Representative images of resected tumors with their respective tumor weight (grams). **D** Tumor volume was increased significantly in transformed Ba/F3–NPM1–TYK2 injected mice but not in Ba/F3-Vector mice. Bars represent the SEM. **E**, **F** In transformed Ba/F3 (NPM1–TYK2) mice, tumor growth was associated with hepatosplenomegaly.

Based on the in vitro transformation potential of the NPM1–TYK2 fusion gene, we further investigated the fusion gene’s tumorigenic potential in an in vivo xenograft model. To assess NPM1–TYK2 tumorigenicity, we subcutaneously injected Ba/F3-Vector or transformed Ba/F3–NPM1–TYK2 cells into the flanks of 5- to 7-week-old female Hsd:Athymic Nude-*Foxn1*^*nu*^ mice (Envigo, Indianapolis, IN). The control mice (six per group) injected with Ba/F3-Vector cells showed no evidence of tumor formation, while the mice (six per group) injected with transformed Ba/F3–NPM1–TYK2 cells developed palpable tumors in 3 days (Fig. 4A, B). Tumor progression was rapid and reached approximately 1400 mm^3^ in size within 15 days (Fig. 4D). The size and weight of lymphoma tumors excised from NPM1–TYK2 mice are shown in Fig. 4C. In addition to tumor growth, Ba/F3–NPM1–TYK2 mice also developed splenomegaly and hepatomegaly, while control mice did not (Fig. 4E, F).

**Fig. 5:**
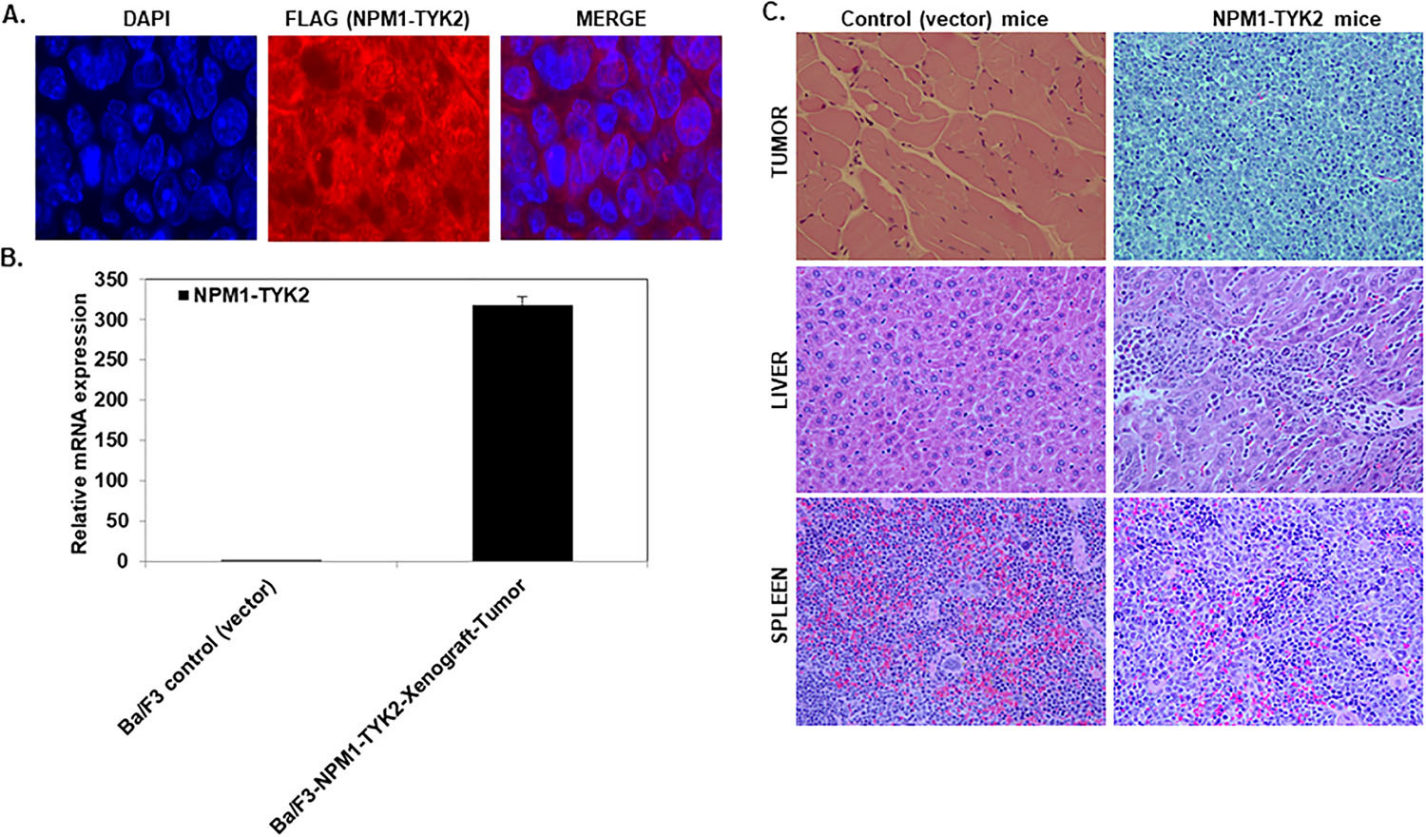
Detection of NPM1–TYK2 in tumors and lymphoma cell infiltration induced hepatosplenomegaly. **A**, **B** Detection of NPM1–TYK2 (FLAG) in tumors by immunostaining and RT-qPCR. Columns represent the mean of three independent experiments; bars represent the SEM. **C** Hematoxylin and eosin (H&E) staining of sections from tumor tissue demonstrate lymphoma cell infiltration into tumor, spleen, and liver.

Different analytical assays confirmed the fusion gene expression at mRNA and protein levels in tumor tissues. Our immunocytochemistry results confirm FLAG (NPM1–TYK2) expression in tumor cells (Fig. 5A). We further analyzed NPM1–TYK2 expression using RT-qPCR to measure fusion gene mRNA transcript levels in tumor tissue (Fig. 5B). Since tumor growth was associated with hepatosplenomegaly, we examined spleen and liver tissue samples of Ba/F3-Vector and Ba/F3–NPM1–TYK2 mice for lymphoma cell infiltration by H&E staining. Immunohistochemistry on the spleen and liver tissue from Ba/F3–NPM1–TYK2 injected mice demonstrated the infiltration of lymphoma cells, but not on tissue from Ba/F3-Vector mice (Fig. 5C).

### Oncogenic NPM1–TYK2 signaling drives in vivo tumorigenicity

**Fig. 6:**
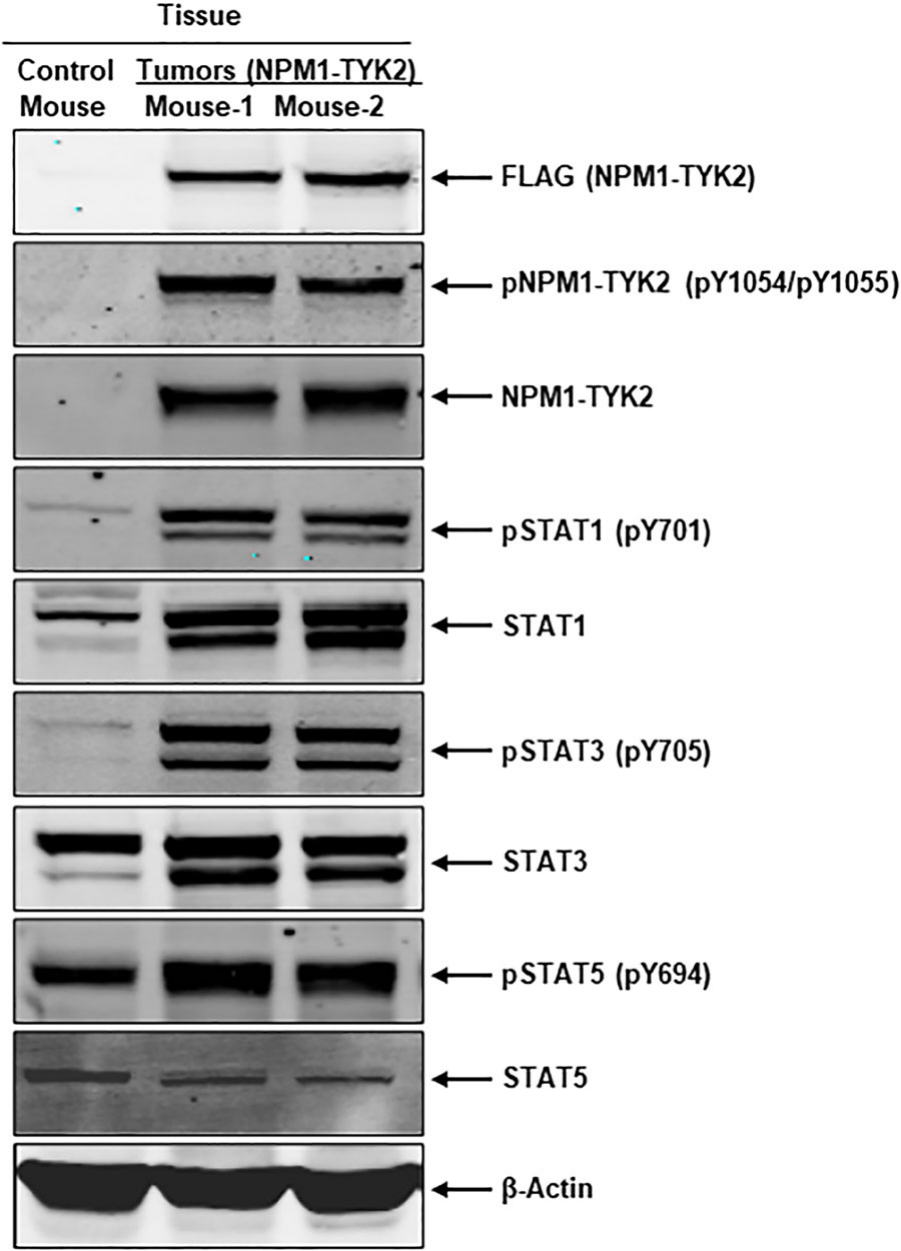
Fusion kinase NPM1–TYK2 constitutive activation drives tumorigenicity in in vivo xenograft models. Representative lysates were made from tissue collected from Ba/F3-Vector and transformed Ba/F3–NPM1–TYK2 injected mice. Western blot analysis was performed for NPM1–TYK2 (FLAG), phospho-NPM1–TYK2, phospho-STAT1/3/5, and respective total proteins. β-Actin served as an internal control.

Upon establishing tumorigenicity in xenograft models, we evaluated the molecular pathways responsible for the fusion gene’s tumorigenic potential. Our studies further focused on exploring the activation of the fusion kinase and upregulation of STAT signaling in xenograft tumors, as observed in in vitro transformation assays. Tissue lysates were prepared from each excised frozen tumor and used for Western blot analysis. Since control mice did not develop tumors, we used respective flank tissue as control tissue to compare with tumor tissues. First, we analyzed NPM1–TYK2 expression levels with FLAG (as the overexpressed NPM1–TYK2 protein is tagged with amino-terminus FLAG epitope) and NPM1–TYK2 antibodies. We also examined phosphorylation of fusion kinase NPM1–TYK2, STAT1, STAT3, and STAT5 and respective total proteins. Results showed NPM1–TYK2 fusion protein expression in tumor tissues with both FLAG and NPM1–TYK2 antibodies, whereas no expression was observed in Ba/F3-Vector mice (Fig. 6). In NPM1–TYK2 tumor tissue, high fusion kinase activity resulted in the upregulation of phosphorylated STAT1, STAT3, and STAT5 (Fig. 6). These signaling data analyses clearly indicate that NPM1–TYK2 signaling mediates tumorigenicity in in vivo models and corroborates the in vitro transformation results.

### NPM1 fusion partner is essential to NPM1–TYK2 mediated oncogenic signaling

**Fig. 7:**
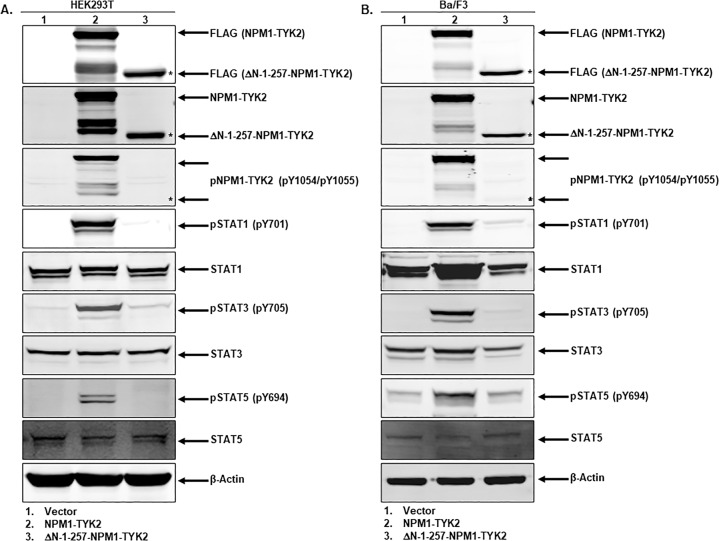
Fusion partner NPM1 is essential for NPM1–TYK2 mediated oncogenic signaling. **A**, **B** Deletion of NPM1 partner in NPM1–TYK2 fusion gene inhibits activation of fusion kinase NPM1–TYK2 mediated signaling in HEK293T and Ba/F3 cells. Western blot analysis of NPM1–TYK2 (FLAG), phospho-NPM1–TYK2, phospho-STAT1/3/5, and respective total proteins. β-Actin served as an internal control.

It is essential to study each fusion partner’s role in oncogenic fusions to better understand disease pathogenesis, ultimately aiding in the development of potential targeted therapeutic interventions^24^. Since NPM1–TYK2 fusion is not fully characterized, here we explored the functional role of the amino-terminus of the NPM1 fusion partner in fusion-driven oncogenicity. To explore the functional role of the NPM1 fusion partner in fusion kinase activity, we deleted the NPM1 partner from full-length NPM1–TYK2 to generate an ΔN-1–257-NPM1–TYK2 deletion construct. We conducted our initial experiments in easy to transfect HEK293T cells. The empty vector, full-length NPM1–TYK2, or ΔN-1–257-NPM1–TYK2 deletion constructs were transfected into HEK293T cells and incubated for 48 h. Total cell lysates were prepared, and Western blot analysis was performed to understand NPM1 deletion effects on NPM1–TYK2 activity. As expected, there was no signal in vector cells (lane 1, Fig. 7A). Full-length NPM–TYK2 (lane 2, Fig. 7A) and NPM1-deletion (lane 3, Fig. 7A) fusion proteins were confirmed by FLAG and NPM1–TYK2 antibodies (Fig. 7A). The full-length fusion protein-induced phosphorylation of NPM1–TYK2 (lane 2, Fig. 7A), but NPM1-deleted fusion protein did not show fusion kinase phosphorylation (lane 3, Fig. 7A). NPM1–TYK2 overexpression induced activation of NPM1–TYK2, resulting in upregulation of STAT1/3/5 signaling as observed in Fig. 2 fusion signaling data. In contrast, deletion of the NPM1 portion from the fusion protein inhibited fusion kinase activity and downstream STAT signaling (lane 3, Fig. 7A). After studying the role of NPM1 fusion partner in HEK293T cells, we made lentiviral particles with ΔN-1–257-NPM1–TYK2 and transduced them into Ba/F3 cells. Stable clones were selected with puromycin and lysates were analyzed for NPM1–TYK2 signaling along with Ba/F3 cells expressing vector and full length-NPM1–TYK2 (Fig. 7B). As observed in HEK293T cells, NPM1 fusion partner deletion from the full-length fusion kinase inhibited NPM1–TYK2 activity and downstream signaling in Ba/F3 cells (Fig. 7B). Together, our results in both HEK293T and Ba/F3 cells exemplify the essential role of the NPM1 fusion partner in NPM1–TYK2 oncogenicity.

### Loss of NPM1 partner abrogates NPM1–TYK2 transformation potential

**Fig. 8:**
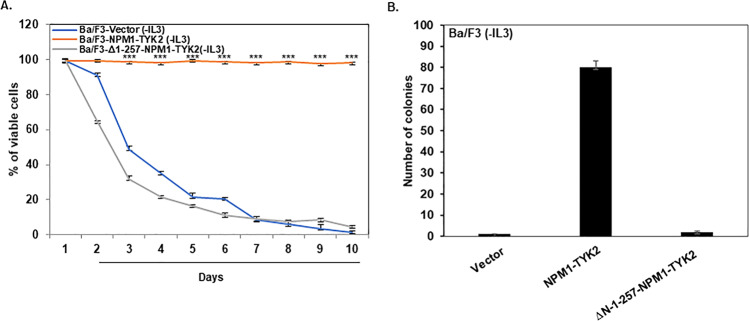
Loss of NPM1 partner abrogates NPM1–TYK2 transformation potential. **A** Deletion of NPM1 portion in NPM1–TYK2 fusion gene abrogates the transformation potential of the NPM1–TYK2 fusion gene in Ba/F3 cells. Ba/F3 cells were transduced with lentiviral particles expressing vector, NPM1–TYK2, and ΔN-1–257-NPM1–TYK2. Cells were grown in RPMI medium without IL-3 growth factor for 10 days. Cell viability from each condition was determined daily by trypan blue exclusion assay. Points represent the mean of three independent experiments; bars represent SEM. ^∗∗∗^*P* < 0.0005 was considered as statistically extremely significant. **B** The deletion of the NPM1 portion in the NPM1–TYK2 fusion gene inhibits the clonogenic potential. Columns represent the mean of three independent experiments; bars represent the SEM.

**Fig. 9:**
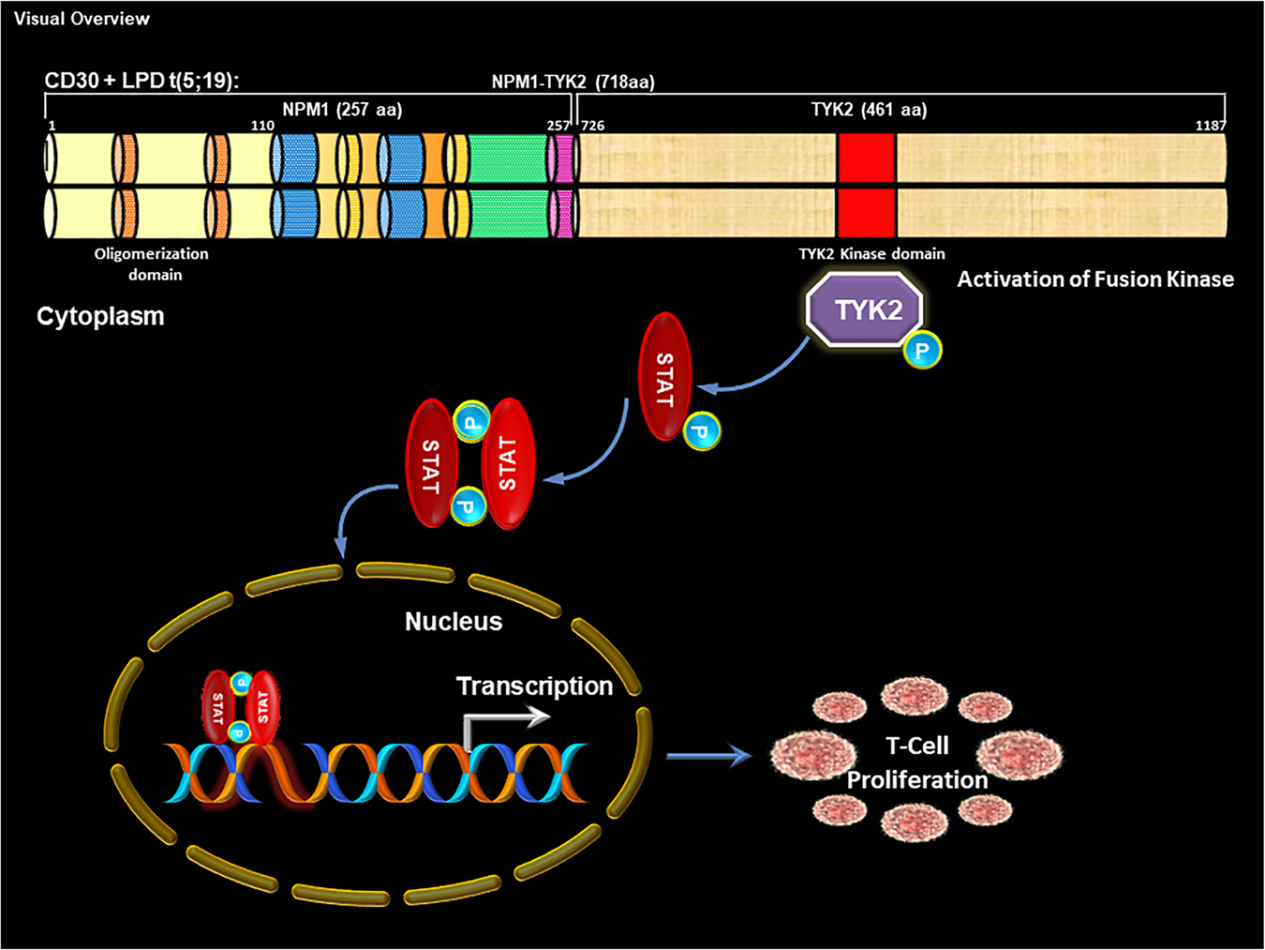
Visual overview. NPM1-TYK2 fusion mediated oncogenic signaling.

Next, we tested the loss of the NPM1 partner’s influence on the viability of Ba/F3 cells. Ba/F3 cells expressing vector, full-length NPM1–TYK2, and ΔN-1–257-NPM1–TYK2 were grown in culture media without IL-3. Cell viability was measured using a trypan blue exclusion assay for ten days. As we observed in previous in vitro viability assays (Fig. 1C), full-length NPM1–TYK2 expressing Ba/F3 cells were viable, whereas vector and NPM1 deletion expressing cells were no longer viable (Fig. 8A). Additionally, we analyzed the proliferation potential of the NPM1–TYK2 fusion without the NPM1 fusion partner. We conducted clonogenic assays on vector, full-length NPM1–TYK2, and ΔN-1–257-NPM1–TYK2 in a Methocult medium without IL-3 growth factor. As shown in Fig. 8B, only the cells expressing full-length NPM1–TYK2 fusion kinase were able to proliferate due to constitutive activation of the fusion kinase and downstream survival signaling (Fig. 9). In contrast, deletion of NPM1 from the fusion kinase resulted in inhibition of both kinase activity and downstream signaling activation ultimately leading to cell death. Overall, these results demonstrate that the NPM1 fusion partner is essential to NPM1–TYK2 mediated clonogenicity.

## Discussion

Existing cancer genomics data has shown that gene fusions derived from chromosomal translocations are frequent oncogenic drivers of many hematologic and solid cancers^25,26^. Targeted therapies are raising hope as effective treatment options for fusion gene-driven maliganancies^27–29^. There has been limited progress in the development of targeted therapies for CTCL due to the lack of understanding of novel genetic alterations and related oncogenic signaling pathways involved in their pathogenesis^30,31^. An earlier study identified NPM1–TYK2 in CD30+ LPDs^7^, but the oncogenic potential of NPM1–TYK2 overexpression on lymphoid cell transformation or tumorigenicity in biological assays had not yet been explored. Therefore, our current studies focused on functional characterization of fusion gene oncogenicity utilizing in vitro and in vivo biological assays.

Understanding the protein subcellular localization is critical due to its influence on diverse cellular processes^32^. In general, wild-type (wt) proteins of NPM1 and TYK2 are localized in the nucleolus and cytoplasm, respectively. Our immunofluorescence data with transformed Ba/F3–NPM1–TYK2 cells showed fusion protein distribution in both nuclear and cytoplasmic compartments. A possible explanation for the nuclear distribution of NPM1–TYK2 is through its heterodimerization with wt-NPM1, as seen in NPM1-ALK expressing ALCL cells^33^.

Malignant cellular transformation is characterized by continuous uncontrolled proliferation^34^. The transformed cells are distinguished from normal counterparts by acquired alterations in growth patterns like anchorage-independent growth potential with no intercellular contact inhibition and xenograft formation^35^. First, we performed in vitro lymphoid cell transformation assays to determine the transformation potential of NPM1–TYK2 using IL-3-dependent lymphoid Ba/F3 cells. The dependency on cytokine signaling of Ba/F3 cell survival is extensively used in hematologic malignancies to understand the oncogenicity of novel kinases, fusion kinases, and downstream signaling of activated tyrosine kinases. Our in vitro transformation assay results distinctly demonstrate that NPM1–TYK2 overexpression leads to the transformation of Ba/F3 cells with survival and proliferation independent of IL-3. Subsequently, our studies focused on identifying the signaling network that is responsible for this transformation potential. In previous fusion kinase oncogenic characterization studies, Ba/F3 cells acquired IL-3-independence through fusion-driven signaling pathways^36^.

TYK2, the first identified JAK family member, is associated with cytokine and growth factor receptors which activate STAT signaling^37^. TYK2 is activated by various cytokines, including numerous interleukins and interferons^38^. TYK2 point mutations found in T-ALL (T-cell acute lymphoblastic leukemia) cell lines exhibited transformation potential via the STAT1/BCL2 pathway^39^. Several activating TYK2 mutations are known to trigger TYK2 signaling, which is essential to the development of T-ALL^39^. Screening of cancer datasets revealed more than fifty TYK2 chromosomal translocations, mostly in hematologic malignancies^40^. As shown in other TYK2 mutated cancers, we investigated NPM1–TYK2 fusion kinase hyperactivation and downstream STAT signaling by Western blot analysis. Our data displayed constitutive phosphorylation of the TYK2 fusion kinase and upregulated downstream effectors STAT1, 3, and 5 in transformed Ba/F3 cells. Furthermore, our NPM1–TYK2–K462R kinase-dead mutant cells showed abrogated signaling and cell viability, confirming that wt-NPM1–TYK2 mediated constitutive activation of fusion kinase is the oncogenic driver responsible for cell transformation.

In vivo xenograft models play an essential role in understanding molecular mechanisms and pathobiology of the disease^41^. Once our in vitro transformation studies characterized NPM1–TYK2 chimera’s oncogenic potential and survival signaling that governs transformation, we conducted in vivo studies using NPM1–TYK2 transformed xenograft models. In line with in vitro transformation, we demonstrated the in vivo tumorigenic potential of the fusion gene in NPM1–TYK2 transformed xenograft models. These mice developed robust tumor progression with associated hepatosplenomegaly. Further immunohistochemistry results revealed infiltration of lymphoma cells in tumors, spleen, and liver. From the excised tumors, signaling data revealed hyperactivation of fusion kinase and upregulation of STAT signaling.

Several fusion proteins involving receptor tyrosine kinases have demonstrated transformation potential in many solid cancers and hematologic malignancies^25^. The majority of these oncogenic fusion kinases contain the carboxyl-terminus kinase and amino-terminus non-kinase partners^42^. The oligomerization sequences of the amino terminus fusion partner are usually responsible for the constitutive activation of carboxyl-terminus kinase partner^24^. Studying the role of each fusion partner helps improve the understanding of disease pathogenesis and directly promotes the development of potential targeted therapeutic interventions. Here, we explored the amino-terminus NPM1 fusion partner’s functional role in fusion kinase oncogenicity. Native NPM1 protein exists as dimeric and oligomeric forms through the amino-terminal oligomerization domain^13,43^. In other NPM1-fusion proteins, the amino-terminus NPM1 fusion partner provides a self-dimerization interface for fusion kinase to form homodimers^14,44^. In our fusion mapping studies, NPM1 deletion from the NPM1–TYK2 fusion gene completely inhibited fusion gene activation and downstream STAT signaling. Further, in vitro transformation results showed that removal of the amino-terminus NPM1 fusion partner abrogated the transformation and clonogenic potential of the fusion kinase. Our results suggest that the NPM1 fusion partner facilitates a homodimerization interface for NPM1–TYK2 which is necessary for its constitutive activation and transformation potential.

In conclusion, our in vitro and in vivo preclinical findings provide functional evidence to identify NPM1–TYK2 as a novel fusion oncogene. Our lymphoid cell transformation and tumorigenicity in xenograft models represent tools necessary to understand the uncovered cellular mechanisms underlying the disease and to assist in future drug screening to develop precision therapies. Importantly, our fusion mapping studies suggest inhibition of NPM1–TYK2 homodimerization by targeting the NPM1 fusion partner could be a potential therapeutic strategy to treat CD30+ LPDs expressing the chimera. Additionally, future preclinical studies focused on inhibition of the TYK2 fusion partner must also be considered in the development of treatment for these fusion-driven lymphomas.

## Methods

### Ethics statement

All animal experiments were approved by the University of Kansas Medical Center institutional animal care and use committee and performed in accordance with relevant regulations and guidelines.

### Cell culture

Ba/F3, SU-DHL-1 (obtained from DSMZ (Deutsche Sammlung von Mikroorganismen und Zellkulturen GmbH, Braunschweig, Germany), HEK293T, and WEHI-3B cell lines were obtained from American Type Culture Collection (ATCC, Manassas, VA, USA). Myla’s cell line was kindly provided by Ryan Wilcox, University of Michigan, USA. HEK293T and WEHI-3B cells were cultured in DMEM medium supplemented with 10% fetal bovine serum (FBS) and 1% penicillin/streptomycin. Myla, SU-DHL-1, and Ba/F3 cells were maintained in RPMI-1640 medium supplemented with 10% FBS and 1% penicillin/streptomycin. Ba/F3 cells were additionally supplemented with a 10% WEHI-3B conditioned medium (as a source of interleukin 3/IL-3), and all stable clones were maintained with 2 µg/ml of puromycin.

### Chemicals and reagents

All reagents and antibodies were purchased from the following: Thermo Scientific, Waltham, MA, USA (RPMI-1640-SH30027; DMEM-SH30081); Corning Life Sciences, USA (Fetal Bovine Serum-MT35010CV, Penicillin/Streptomycin-30-002-CI); InvivoGen, San Diego, CA, USA (Puromycin-58-58-2); STEMCELL Technologies, Vancouver, BC, Canada (MethoCult-H4100 −04100); Cell Signaling Technology, Beverly, MA, USA (TYK2/NPM1–TYK2-14193, phospho-TYK2/phospho-NPM1–TYK2-9321, pSTAT3-9145, STAT5-9363, STAT1-14994); BD Biosciences San Jose, CA, USA (STAT1-610185, phospho-STAT1-612132, STAT3-610189, Phospho-STAT5-611964, β-Actin-612656); Sigma-Aldrich, St. Louis, MO, USA (FLAG-F3165, Trypan Blue-T8154).

### Cloning and generation of stable Ba/F3–FLAG–NPM1–TYK2 and Ba/F3–FLAG-∆1–257–NPM1–TYK2 cell lines

Human full-length NPM1–TYK2 cDNA^7^ was amplified by PCR. The PCR product was then gel purified, digested with XbaI and NheI restriction enzymes, and subcloned into a lentiviral plasmid pCDH-EF1-MCS-T2A-Puro vector (purchased from System Biosciences, Palo Alto, CA, USA). The colonies were screened, and positive clones were confirmed by Sanger DNA sequencing. The lentiviral plasmids, either empty vector (pCDH-EF1-MCS-T2A-Puro) or recombinant plasmid (pCDH-EF1-FG-NPM1–TYK2) were transfected along with packaging plasmid (psPAX2) and envelop plasmids (pMD2.G) into HEK293T cells using Fugene HD transfection reagent (Roche Applied Science). The lentiviral particles were collected after 48 h. Ba/F3 cells were transduced with either empty vector or pCDH-EF1-FG-NPM1–TYK2 lentivirus and the selection was carried out after 48 h with 2 µg/ml puromycin. Ba/F3-pCDH-vector cell growth was IL-3-dependent, while the transformed Ba/F3–FG–NPM1–TYK2 cell growth was IL-3 independent. The fusion gene NPM1–TYK2 mRNA expression levels were confirmed by qPCR and protein expression levels were confirmed by Western blotting using FLAG and NPM1–TYK2 antibodies. The deletion constructs Ba/F3–FG–∆N-1–257–NPM1–TYK2 were generated from NPM1–TYK2 cDNA using primers to amplify the amplicon spanning only the TYK2 portion. The clones were screened, and positive clones were confirmed by Sanger DNA sequencing. Lentiviral particles were made, as explained above. The deletion constructs protein expression levels were confirmed by Western blotting for FLAG and NPM1–TYK2. Stable cells expressing the deletion construct were IL-3 dependent due to the loss of the NPM1 fusion partner.

### Generation of NPM1–TYK2 kinase-dead mutant stable cell line

FG-NPM1–TYK2-K462R kinase-dead mutant was created by performing Site-Directed Mutagenesis using site-specific mutagenesis primers (FP 5′-ACTGGCGAGATGGTGGCGGTGCGGGCCCTCAAGGCAGACTGCGGC-3′ and RP 5′-GCCGCAGTCTGCCTTGAGGGCCCGCAC CGCCACCATCTCGCCAGT-3′) and wt-NPM1–TYK2 cDNA as a template (Q5 site-directed mutagenesis kit, New England BioLabs Inc, Ipswich, Massachusetts, USA)^7^. The mutant sequence was confirmed by Sanger sequencing. Ba/F3 cells were transduced with FG–NPM1–TYK2–K462R lentiviral particles and stable clones were established using puromycin antibiotic selection. Ectopic kinase-dead fusion protein expression levels were confirmed by Western blot using FLAG antibody.

### Western blot analysis

The cells were harvested, washed with phosphate-buffered saline (PBS), added in lysis buffer (25 mM Tris. HCl, 150 mM NaCl, 25 mM NaF, 0.5 mM Na-orthovanadate, 1% Triton-X, 1 mM Benzamidine) with protease and phosphatase inhibitors and incubated on ice for 20 min. Cell lysates were centrifuged at 10,000 RPM for 15 min, and protein concentrations were determined using the BCA protein assay kit (Pierce Biotechnology, Rockford, IL USA). The protein samples were resolved by sodium dodecyl sulfate-polyacrylamide gel electrophoresis and immunoblotting was performed. The blots were scanned using the Odyssey IR scanner (Li-COR Biosciences, Lincoln, NE, USA). All blots derive from the same experiment and were processed in parallel.

### Immunofluorescence

To analyze the cellular distribution of NPM1–TYK2, Ba/F3–FG–NPM1–TYK2 cell slides were prepared by cytospin technique. Similarly, Ba/F3-pCDH-vector cells were also processed and used as a control. Both control and FG–NPM1–TYK2 overexpressed cells were fixed with 4% paraformaldehyde and permeabilized with methanol/glacial acetic acid (3:1). After fixation, the cells were incubated with FLAG primary antibody overnight, washed with PBS, and then incubated with secondary antibody conjugated with Dylight-594 for an hour (D1-2594, Vector Laboratories, Burlingame, CA). Coverslips were mounted with Vectashield antifade mounting medium containing DAPI (4′, 6′-diamidino-2-phenylindole, Vector laboratories, Burlingame, CA). The images were acquired using an Eclipse E1000 microscope (Nikon).

### Immunohistochemistry

Paraffin-embedded tissues were sectioned at 4 µM size and mounted on the glass slide. Sections were dewaxed in xylene overnight and rehydrated through in gradient ethanol series, followed by washing with PBS. Hematoxylin staining was performed using Leica Surgipath SelecTech Hematoxylin. This is followed by brief immersion in Richard-Allan Scientific Eosin-y with Phloxine, then dehydration with reagent alcohol, xylene, and mounted with a coverslip using permanent mounting media. The slides were examined with Nikon Eclipse E1000 microscope under a 40× objective.

### RT-qPCR

#### Reverse transcription

Total RNA was isolated using an RNA isolation kit (Roche Applied Bioscience) and reverse transcribed with High-Capacity cDNA reverse transcription kit (Applied Biosystems) according to the manufacturer’s protocol. The resulting cDNA was diluted and used in SYBR green qPCR assay.

#### SYBR green qPCR assay

qPCR was performed using Power SYBR Green Mastermix (Applied Biosystems, Carlsbad, CA, USA) on an Applied Biosystems StepOne Plus Real-Time PCR System. All oligonucleotide primers were obtained from Integrated DNA Technologies (Coralville, IA). All assays were performed in triplicates and repeated twice, and results were plotted as average fold change relative to GAPDH. Primers used for validation of NPM1–TYK2 fusion transcripts in cell lines by SYBR green assay using the following pair of forward and reverse primers; 5′-ACTCAAAACCATCATCAACACCA-3′; 5′-GTTCCGGCCACACACATTAC-3′.

### Clonogenic assay

The clonogenic assays were performed with Ba/F3 cells to determine the oncogenic potential of ΔN-1–257-NPM1–TYK2 in comparison to full-length NPM1–TYK2 along with vector control. Stable Ba/F3 cells were grown in Methocult media without IL-3 in triplicates. The cells were allowed to grow and form colonies for seven days. The colonies were counted and presented as percentages.

### Cell viability

To compare the oncogenic mechanisms of the full-length NPM1–TYK2 and ∆N-1–257-NPM1–TYK2, resulting in the survival of Ba/F3 cells, we used trypan blue exclusion assays, and cell viability was represented by a percentage. Further, we conducted similar viability experiments with NPM1–TYK2–K462R kinase-dead mutant cells to validate the fusion kinase-driven transformation potential.

### Animal experiments

Upon confirming in vitro transformation potential of the NPM1–TYK2 fusion gene, we next aimed to characterize the in vivo tumorigenic potential of a novel fusion gene utilizing an NPM1–TYK2-transformed Ba/F3-xenograft model. We used five to seven-week-old female Hsd:Athymic Nude-Foxn1nu mice (Envigo, Indianapolis, IN) to validate the tumorigenic potential of fusion kinase NPM1–TYK2. We injected 5 million Ba/F3-vector control and Ba/F3–FG–NPM1–TYK2 cells in the flank via a subcutaneous route (six mice in each group). Tumor size was measured by the Vernier caliper and tumor volumes were calculated using the modified ellipsoid formula: *V* = 1/2(length × width^2^). The mice were euthanized when the tumor size reached 2000 mm^3^ or the tumors became necrotic. Tumor tissues, spleen, and liver were collected from euthanized mice to perform Western blot analysis for signaling and hematoxylin and eosin (H&E) staining to evaluate lymphoma cell infiltration associated with hepatosplenomegaly.

### Statistical analysis

Statistical analysis was performed using Microsoft Excel. Significant differences were determined using the Student’s *t*-test.

### Reporting summary

Further information on research design is available in the Nature Research Reporting Summary linked to this article.

## Supplementary information

Unprocessed blots

REPORTING SUMMARY

## Data Availability

The authors declare that all relevant data for this study are included within the paper. For any additional information regarding the supporting data, please contact the corresponding author with a reasonable request.
