# Supplementary figures and images for: Functional characterization of NPM1–TYK2 fusion oncogene
Source: NPJ Precis Oncol. 2022 Jan 18;6:3. doi: 10.1038/s41698-021-00246-4 (PMC8766497; doi:10.1038/s41698-021-00246-4)

Figure 2.

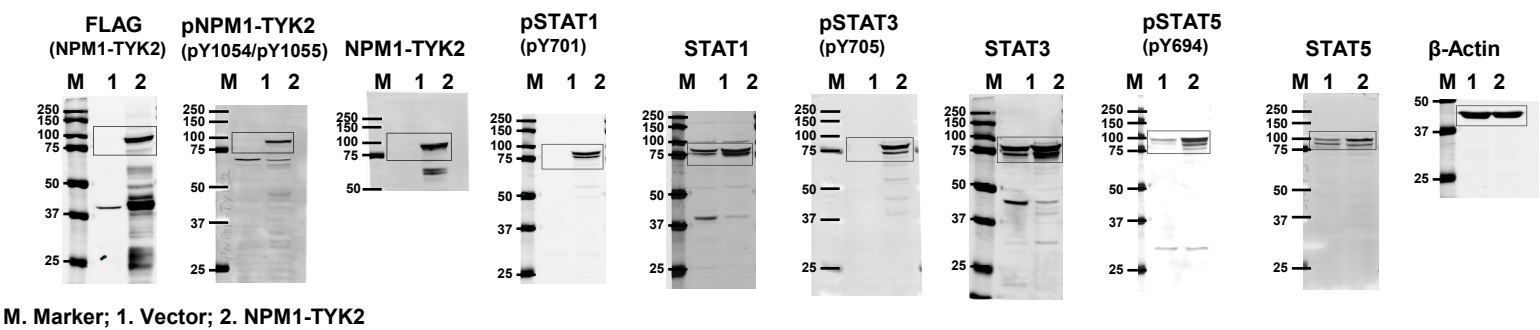

Figure 3.

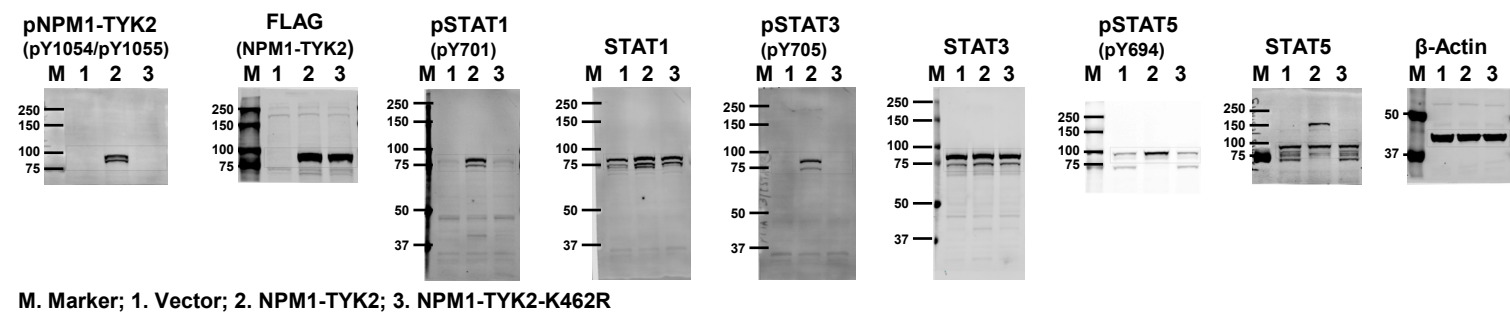

Figure 6.

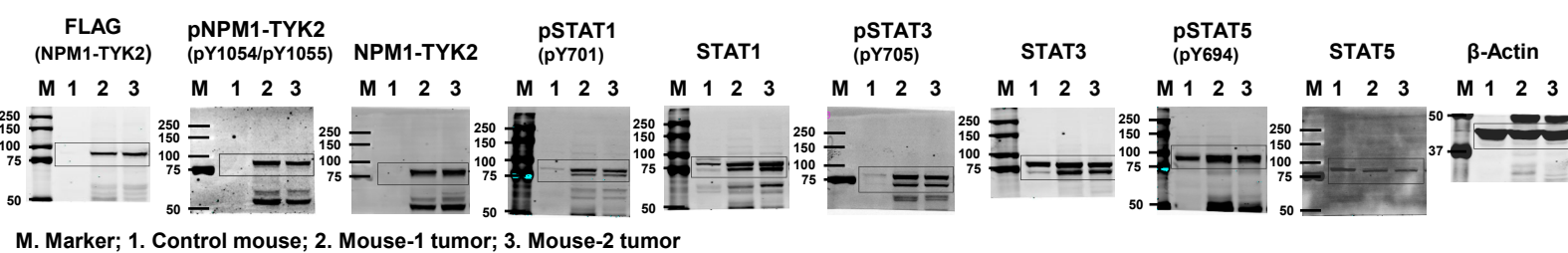

Figure 7A.

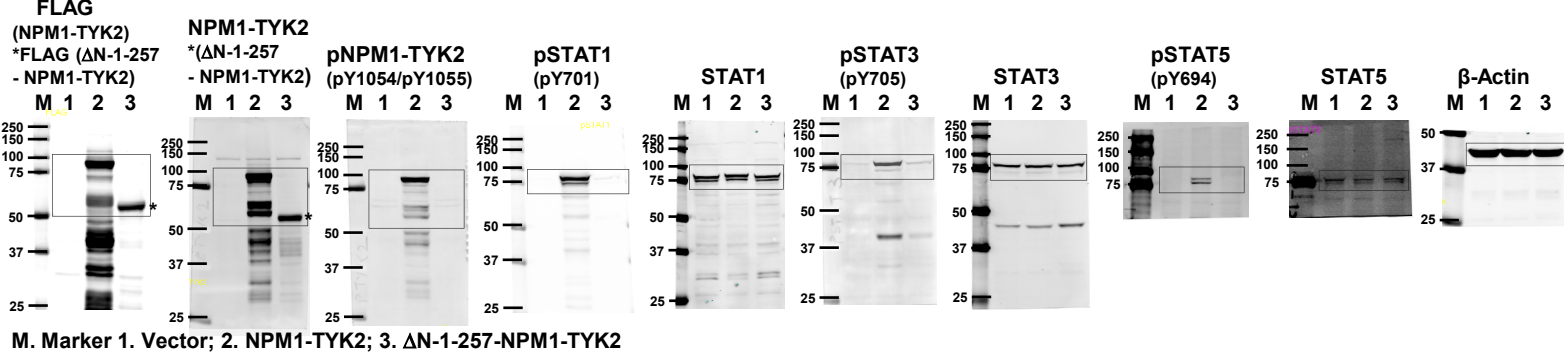

Figure 7B.

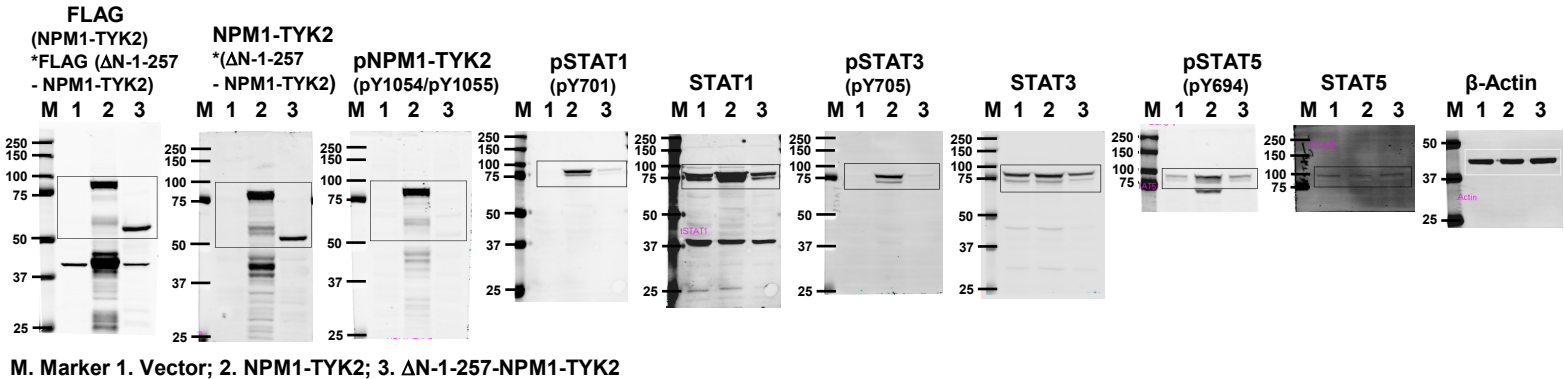

Supplement: Supplementary file 1 — Unprocessed blots [file 41698_2021_246_MOESM1_ESM.pdf]
