# Supplementary material for: Functional characterization of NPM1–TYK2 fusion oncogene
Source: NPJ Precis Oncol. 2022 Jan 18;6:3. doi: 10.1038/s41698-021-00246-4 (PMC8766497; doi:10.1038/s41698-021-00246-4)
Supplement: Supplementary file 2 — REPORTING SUMMARY [file 41698_2021_246_MOESM2_ESM.pdf]

nature portfolio

Corresponding author(s): Ramesh Balusu

Last updated by author(s): 2021/10/29

Reporting Summary

Nature Portfolio wishes to improve the reproducibility of the work that we publish. This form provides structure for consistency and transparency in reporting. For further information on Nature Portfolio policies, see our Editorial Policies and the Editorial Policy Checklist.

Statistics

For all statistical analyses, confirm that the following items are present in the figure legend, table legend, main text, or Methods section.

n/a	Confirmed	
☐	☒	The exact sample size (n) for each experimental group/condition, given as a discrete number and unit of measurement
☐	☒	A statement on whether measurements were taken from distinct samples or whether the same sample was measured repeatedly
☐	☒	The statistical test(s) used AND whether they are one- or two-sided<br>*Only common tests should be described solely by name; describe more complex techniques in the Methods section.*
☒	☐	A description of all covariates tested
☒	☐	A description of any assumptions or corrections, such as tests of normality and adjustment for multiple comparisons
☐	☒	A full description of the statistical parameters including central tendency (e.g. means) or other basic estimates (e.g. regression coefficient) AND variation (e.g. standard deviation) or associated estimates of uncertainty (e.g. confidence intervals)
☐	☒	For null hypothesis testing, the test statistic (e.g. F, t, r) with confidence intervals, effect sizes, degrees of freedom and P value noted<br>*Give P values as exact values whenever suitable.*
☒	☐	For Bayesian analysis, information on the choice of priors and Markov chain Monte Carlo settings
☒	☐	For hierarchical and complex designs, identification of the appropriate level for tests and full reporting of outcomes
☒	☐	Estimates of effect sizes (e.g. Cohen's d, Pearson's r), indicating how they were calculated

Our web collection on statistics for biologists contains articles on many of the points above.

Software and code

Policy information about availability of computer code

Data collection	N/A
Data analysis	N/A

For manuscripts utilizing custom algorithms or software that are central to the research but not yet described in published literature, software must be made available to editors and reviewers. We strongly encourage code deposition in a community repository (e.g. GitHub). See the Nature Portfolio guidelines for submitting code & software for further information.

Data

Policy information about availability of data

All manuscripts must include a data availability statement. This statement should provide the following information, where applicable:

- Accession codes, unique identifiers, or web links for publicly available datasets
- A description of any restrictions on data availability
- For clinical datasets or third party data, please ensure that the statement adheres to our policy

The authors declare that all relevant data for this study are included within the paper. For any additional information regarding the supporting data, please contact the corresponding author with a reasonable request

Field-specific reporting

Please select the one below that is the best fit for your research. If you are not sure, read the appropriate sections before making your selection.

☒ Life sciences ☐ Behavioural & social sciences ☐ Ecological, evolutionary & environmental sciences

For a reference copy of the document with all sections, see nature.com/documents/nr-reporting-summary-flat.pdf

Life sciences study design

All studies must disclose on these points even when the disclosure is negative.

Sample size	For animal experiments, n=6 mice/group.
Data exclusions	There were no data exclusions.
Replication	Cell based assays performed two or more independent experiments conducted in triplicates.<br>Animal experiments: Tumor sizes were measured during the time period and weight of the tumors at the end of the experiment.
Randomization	Animal experiments conducted with two distinct (control and fusion gene) groups.
Blinding	In animal experiments, tumor growth was measured by technician.

Reporting for specific materials, systems and methods

We require information from authors about some types of materials, experimental systems and methods used in many studies. Here, indicate whether each material, system or method listed is relevant to your study. If you are not sure if a list item applies to your research, read the appropriate section before selecting a response.

Materials & experimental systems

n/a	Involved in the study	
☐	☒	Antibodies
☐	☒	Eukaryotic cell lines
☒	☐	Palaeontology and archaeology
☐	☒	Animals and other organisms
☒	☐	Human research participants
☒	☐	Clinical data
☒	☐	Dual use research of concern

Methods

n/a	Involved in the study	
☒	☐	ChIP-seq
☒	☐	Flow cytometry
☒	☐	MRI-based neuroimaging

Antibodies

Antibodies used	Cell Signaling Technology, Beverly, MA, USA (TYK2/NPM1-TYK2-14193, phospho-TYK2/phospho-NPM1-TYK2-93213, pSTAT3-9145, STAT5-9363, STAT1-14994); BD Biosciences San Jose, CA, USA (STAT1-610185, phospho-STAT1-612132, STAT3-610189, Phospho-STAT5-611964, β-Actin-612656); Sigma-Aldrich, St. Louis, MO, USA (FLAG-F3165). All antibodies were used at 1:1000 dilution.
Validation	Antibodies purchased from Cell Signaling Technology, BD Biosciences, and Sigma-Aldrich. All these antibodies routinely used in the lab. Each antibody validated by Western blotting as per manufacturer's instructions.

Eukaryotic cell lines

Policy information about cell lines

Cell line source(s)	Ba/F3 cells (DSMZ), SU-DHL-1 (DSMZ), HEK293T (ATCC), WEHI-3B (ATCC), Myla cell line was kindly provided by Ryan Wilcox, University of Michigan, USA.
Authentication	All authenticated cell lines purchased from ATCC and DSMZ, initial passages were used in all the experiments. Myla cell line was kindly provided by Ryan Wilcox, University of Michigan, USA. Myla and other cell lines were further validated by qPCR for fusion transcript and fusion protein by Western blot analysis.
Mycoplasma contamination	All cell lines are negative for mycoplasma contamination.
Commonly misidentified lines<br>(See ICLAC register)	N/A

Animals and other organisms

Policy information about studies involving animals; ARRIVE guidelines recommended for reporting animal research

Laboratory animals	Five to seven-week old female Hsd:Athymic Nude-Foxn1nu mice (Envigo, Indianapolis, IN)
Wild animals	No wild animals used
Field-collected samples	No field-collected samples used
Ethics oversight	University of Kansas Medical Center institutional animal care and use committee

Note that full information on the approval of the study protocol must also be provided in the manuscript.
